# GERONTOLOGIC BIOSTATISTICS: MERGING WITH DATA SCIENCE AND TOWARD PERSONALIZED MEDICINE

**DOI:** 10.1093/geroni/igac059.708

**Published:** 2022-12-20

**Authors:** Michelle Shardell, Terrence Murphy, Heather Allore

## Abstract

The sub-discipline of gerontologic biostatistics (GBS) was introduced in 2010 to emphasize the special challenges encountered in the design and analysis of research studies of older persons. These challenges center on the multifactorial nature of human aging, characterized by the parallel and progressive deterioration of diverse organ and cellular systems that eventually results in death. Ten years after the introduction of GBS, which initially focused on important aspects of design and analysis that ensure their statistical validity, we update how GBS has been enriched by evolving practices. We present individual sessions on three seminal developments in the practice of GBS: integration of data science and multiple streams of data, including those automated and or multidisciplinary in nature; enhanced methods of addressing the heterogeneity of treatment effects from health-related interventions for older patients; and how interactive visualization can help specific patients locate themselves along the continuum of individualized treatment effects. We conclude our presentation with a session that reviews three prominent trends in the validation of the heterogeneity inherent to the assessment of health among older adults. Reflecting this era of big gerontological data, we discuss several established modeling approaches for validation, the proliferation of signal intensive behavior phenotypes, and the deep characterization of phenotypes through OMICS studies and multimodal approaches. All talks discuss pitfalls and areas of future development and draw from published studies. We are submitting as an interest group collaborative panel submission between two interest groups: Epidemiology of Aging and Measurement, Statistics and Design.

